# Factors Impacting Primary Care Engagement in a New Approach to Integrating Care in Ontario, Canada

**DOI:** 10.5334/ijic.5704

**Published:** 2022-03-04

**Authors:** Amanda C. Everall, Kristina M. Kokorelias, Shannon L. Sibbald, Walter P. Wodchis, Gayathri Embuldeniya

**Affiliations:** 1 Health System Performance Network, Institute of Health Policy, Management, and Evaluation, University of Toronto, Toronto, Ontario, Canada; 2 St John’s Rehab, Sunnybrook Research Institute, Sunnybrook Health Sciences Centre, Toronto, Ontario, Canada; 3 School of Health Studies, Faculty of Health Sciences, Department of Family Medicine, Western University, London, Ontario, Canada; 4 Institute for Better Health, Trillium Health Partners, Mississauga, Ontario, Canada

**Keywords:** primary care engagement, primary care physicians, integrated care, healthcare providers, qualitative interviews, health system transformation

## Abstract

**Introduction:**

In 2019, Ontario’s Ministry of Health (the Ministry) introduced Ontario Health Teams (OHTs) to provide population-based integrated healthcare. Primary care was foundational to this approach. We sought to identify factors that impacted primary care engagement during OHT formation from different perspectives.

**Methods:**

Interviews with 111 participants (administrators n = 80; primary care providers n = 17; patient family advisors = 14) from 11 OHTs were conducted following a semi-structured guide. Interviews were transcribed, coded, and thematically analyzed.

**Results:**

Participants felt that primary care engagement was an ongoing, continuous cycle. Four themes were identified: 1) ‘A low rules environment’: limited direction from the Ministry (system-level), 2) ‘They’re at different starting points’: impact of local context (initiative-level); 3) ‘We want primary care to be actively involved’: engagement efforts made by OHTs (initiative-level); 4) ‘Waiting to hear a little bit more’: primary care concerns about the OHT approach (sector-level). Thirteen factors impacting primary care engagement were identified across the four themes.

**Discussion and Conclusion:**

The 13 factors influencing primary care engagement were interconnected and operated at health system, integrated care initiative, and sector levels. Future research should focus on integrated care initiatives as they mature, to address potential gaps in the involvement of primary care physicians.

## Introduction

Health systems are shifting towards more integrated approaches to care [1,2]. To support integrated care within the Canadian province of Ontario, the Ministry of Health (the Ministry) introduced Ontario Health Teams (OHTs) [3]. At maturity, OHTs will be:

*“…groups of healthcare providers and organizations that are clinically and fiscally accountable for delivering a full and coordinated continuum of care to a defined geographic population.” (p.1)* [3]

OHTs include multiple healthcare sectors, from primary care to social services [3] and aim to increase connectivity between health services across care settings by providing care through a single, cohesive team and improve outcomes linked to the quadruple aim: patient and population health outcomes, patient/caregiver experiences, provider experiences, and overall cost [4]. OHTs are currently in the developmental stages of integration, focusing on self-identified priority populations within their geographic area. The structure and aims of OHTs fit within the World Health Organization’s (WHO) working definition of integrated care:

*“a concept bringing together inputs, delivery, management and organization of services related to diagnosis, treatment, care, rehabilitation and health promotion. Integration is a means to improve the services in relation to access, quality, user satisfaction and efficiency.”* [1]

As of September 2021, 50 OHTs have been approved [5]. The Ministry provided OHTs with guidance documents and resources to assist with their formation [3]; however, very little was mandated by the Ministry (e.g., identification of a specific initial priority population). Instead, the Ministry encouraged the OHTs to establish their own governance structure, find local solutions to the delivery and management of healthcare, and leverage existing partnerships [6,7,8,9,10] to capitalize on existing strengths [4]. Existing partnerships within some OHTs were developed through prior integrated care initiatives such as Health Links [9] and Integrated Funding Models [10], which both included partnerships with primary care physicians (PCPs). However, the Ministry explicitly called for improved involvement of PCPs in a patient’s healthcare journey [4].

Primary care has been viewed as foundational to Canada’s health system and the Ministry described primary care physicians as “key to the success [and] essential cornerstones” of the OHT model [4]. Within the OHT initiative, PCP engagement entails the provision of primary care services under the OHT umbrella as well as their contribution at decision-making tables. More recently, PCP engagement has been identified as a key principle for successful health systems integration [2,11] and engaging physicians through communication and feedback has been found to be key in engagement efforts [12]. Case studies in seven countries suggest that a lack of primary care engagement in most initiatives may be due to the hesitancy of primary care providers to share data about their patients and to play a proactive role in care delivery [13]. Other barriers to PCP engagement in integrated care initiatives have included availability of primary care physicians, reimbursement, legal liabilities and accountabilities, information technology, complexity of chronic disease management, as well as local contextual factors [14,15].

While practices for physician engagement [16,17,18] and early lessons learned from OHTs have been published [19], there is a lack of data focusing on factors influencing PCP engagement in organisational improvement work [18]. There is also a dearth of data examining how non-PCP stakeholders understand issues of relevance to primary care. Therefore, our aim was to identify factors affecting primary care engagement from different lenses during the formation of the OHT initiative in Ontario, Canada.

## Methods

### Study Design

We conducted a descriptive qualitative study [20]. The study was approved by the research ethics board of the University of Toronto.

### Participants

Eleven OHTs were selected randomly from 30 applicant teams to capture a representative sample across geography (urban/suburban vs. rural) and lead sector (hospital vs. other). All OHTs that were contacted by the research team agreed to participate in the study. OHTs supplied the research team with a list of key interviewees across a range of health care sectors, who had a significant role in the formation of the OHT. Participants included PCPs and nurse practitioners, as well as administrative leaders (e.g., hospital CEOs, project managers, OHT leads, organizational leads), and patient and family advisors (PFAs).

### Data Collection

Semi-structured interviews were conducted between January and March 2020 to explore the process of early OHT formation, including strategies, challenges, facilitators, and the processes involved in care transformation (see Appendix A). The interviews were completed by five trained members of the research team. They averaged 60 minutes and were conducted via telephone or videoconference, depending on participant preference. Participants provided verbal consent prior to their interviews. Interviews were audio recorded, professionally transcribed verbatim, and checked for accuracy.

### Data Analysis

Our thematic analysis followed the six steps outlined by Braun and Clarke [21]. To begin the analysis, a selection of transcripts was open-coded by all members of the research team. First impressions and reoccurring concepts were noted and became the basis for an exhaustive codebook with definitions and examples. The codebook was then applied to another selection of transcripts and refined by the research team to ensure consistent application of codes and to ensure all concepts were captured. Over three months, the research team that had conducted the interviews coded all transcripts guided by the codebook using NVivo 12 [22]. Data from codes relevant to PCP engagement were reviewed by the team during bi-weekly meetings to identify factors and discuss thematic groupings. As themes were proposed, the team considered alternate perspectives until consensus was reached. ACE, KMK, and SLS met to clarify and refine the themes, with input from GE and WPW.

## Results

In total, 111 participants were interviewed, including administrators (e.g., project managers, OHT leads, organizational leads, etc.; n = 80), PFAs (n = 14), and PCPs (n = 17) across 11 OHTs (see ***Table 1***). Most PCPs were employed in a primary care team-based setting (e.g., Family Health Team [23] or Community Health Center [24]) rather than as an independent practitioner. PCPs were mostly male (n = 14) and were generally mid-to-late career family physicians.

**Table 1:** Characteristics of Ontario Health Teams and Summary of Interview Participants.


OHT ID	GEOGRAPHY	LEAD ORGANIZATION	ADMINISTRATOR PARTICIPANTS (N)	PFA PARTICIPANTS (N)	PCP PARTICIPANTS (N)	TOTAL PARTICIPANTS (N)

OHT01	Rural	Hospital	7	1	2	10

OHT06	Urban/Suburban	Hospital	8	1	3	12

OHT07	Urban/Suburban	Non-Hospital	6	2	1	9

OHT15	Rural	Non-Hospital	7	2	1	10

OHT16	Urban/Suburban	Hospital	7	1	2	10

OHT20	Urban/Suburban	Hospital	8	3	1	12

OHT23	Rural	Hospital	7	1	2	10

OHT24	Urban/Suburban	Non-Hospital	6	0	1	7

OHT25	Urban/Suburban	Hospital	10	1	1	12

OHT27	Urban/Suburban	Hospital	7	1	2	10

OHT28	Urban/Suburban	Hospital	7	1	1	9

**Total**			**80**	**14**	**17**	**111**


OHT = Ontario Health Team, PFA = Patient and family advisors, PCP = Primary care physician.

Participants from most OHTs described primary care engagement during the formative phase as an ongoing, continuous cycle. Both administrators and PCPs agreed that primary care engagement was the “*key to the success*” (OHT15_5, Administrator) of the OHT approach; however, some divergence in views between PCPs and administrators was evident relating to factors affecting PCP engagement. This divergence was emphasized through shifting pronouns showcased in participant quotes (‘us’ versus ‘them’) and provides a window into how participant’s assumptions, biases, and sympathies could also inform divergent perspectives.

Four key themes emerged. The first theme describes health system-level factors: 1) ‘A low rules environment’: limited direction from the Ministry. Two themes described initiative-level factors: 2) ‘They’re at different starting points’: impact of local context, and 3) ‘We want primary care to be actively involved’: engagement efforts made by OHTs. The last theme describes sector-level factors: 4) ‘Waiting to hear a little bit more’: primary care concerns about the OHT approach. Factors affecting primary care engagement within each theme are identified and explored from the viewpoint of PCPs, administrators, and when applicable, PFAs (see ***Table 2***).

**Table 2:** Themes and Factors Affecting Primary Care Engagement.


LEVEL	THEME	FACTORS

System	*‘A low rules environment’*: limited direction from the Ministry	- Grassroots, localized approaches

Initiative	*‘They’re at different starting points’*: impact of local context	- State of primary care - Geographical location - History of collaboration - Technology

	*‘We want primary care to be actively involved’*: engagement efforts made by the OHTs	- PCP outreach and engagement efforts - Timing of PCP engagement - Capacity of PCP involvement

Sector	*‘Waiting to hear a little bit more’*: primary care concerns about the OHT approach	- Remuneration - Time commitment and timing of meetings - Ensuring representation - Professional autonomy - Impact on daily practice


### Theme 1 – ‘A low rules environment’: limited direction from the Ministry

#### Grassroots, localized approaches

Ontario’s Ministry purposefully emplaced OHTs within a “*low rules environment*” (OHT27_3, Administrator) to permit grassroots, localized approaches to integrated care. While most administrators and some PCPs approved of this approach, the lack of direction from the Ministry was described as a foundational challenge to primary care engagement. PCPs often felt that the supports and direction coming from the Ministry were insufficient:

*“…they’re [primary care] very interested in this but […] they need other things including other support from the Ministry if they’re going to actually change their model in some way…” (OHT06_6, PCP)*

Some PCPs felt that this lack of direction was indicative of insufficient system-level commitment to the approach and wanted more certainty before joining the initiative. PCPs also doubted the sustainability of the OHT initiative given the political nature of healthcare decisions in Ontario, questioning “*if there’s government change, are we suddenly going to stop?*” (OHT01_1, PCP). Administrators felt that “*there’s sort of a ‘prove it’*” mentality (OHT20_6, Administrator) among physicians, whereby PCPs placed the onus on the OHT and the government to clearly explain the changes that would be made to primary care and describe how these changes would improve workflow and patient care.

### Theme 2 – ‘They’re at a different starting point’: impact of local context

The regional context in which each OHT was situated impacted primary care engagement efforts. Factors included the fragmented state of primary care, geographical location and catchment area size, history of cross-sectoral collaboration and integration, and technology.

#### State of primary care

The variability of local primary care practice models and organization in Ontario was described by both PCPs and administrators in most OHTs as a barrier to primary care engagement, as “*every OHT will look different according to the way that their physicians are organized*” (OHT01_2, Administrator). Primary care physicians were described as “*not a monolithic group*” (OHT28_10, Administrator) and administrators felt that primary care physicians who were either affiliated with a primary care group (e.g., Family Health Teams or Organizations) or directly associated with the signatory hospital were easier to engage because there were often prior communication channels or pre-existing relationships.

In OHTs where most PCPs were independent practitioners, administrators faced substantial barriers to outreach and engagement (e.g., lacking a comprehensive list of practitioners in their catchment area, limited relationships). Participants felt that differing models of primary care in each OHT required a local approach because “*the strategies and the starting points are different*” (OHT16_7, Administrator).

#### Geographical location

Participants’ experiences were also often influenced by their geographical location and the size of their catchment area. For example, more rural areas seemed to have stronger cross-sectoral relationships with primary care:

*“I cannot emphasize enough the difference between these small community relationships and maybe [Large Urban Center], right. Everybody works together. The physicians wear multiple hats. They’re primary care providers, and they do hospitalist work… they’re the anesthetists in the hospital, and they look after patients in the long-term care homes, and they look after the patients in the prison.” (OHT15_2, Administrator)*

In OHTs with larger catchment areas, participants described challenges with the OHT attribution model (e.g., the desire to provide a full and coordinated continuum of care for a specific population) within a geographic region, especially when patients and primary care providers overlapped in multiple OHT catchment areas. Indeed, the attribution model for primary care in urban centers excluded some PCPs who were signatories on their preferred OHT, leading to confusion among PCPs and administrators over where PCPs belonged.

#### History of collaboration

History of collaboration and integration, or lack thereof, was felt to be a key factor impacting primary care engagement by both PCPs and administrators. OHTs with previous successful collaborative and integrated care initiatives were generally more successful with primary care engagement, whereas regions with historic systemic disenfranchisement, for example, with the main hospital, experienced challenges.

*“There was a real break between the family physicians and the hospital, you know dating back years.” (OHT24_1, Administrator)*

Some physicians also had experienced unsuccessful system integration initiatives within the province. Often, the OHT approach was considered an avenue to amend these relationships and improve the state of collaboration and integration across sectors.

While not pervasive, some administrators across OHTs hinted at a challenging history of collaboration with PCPs and felt that they were often hesitant to change. Indeed, physicians described themselves as traditional, cautious, and “*a tricky bunch*” (OHT01_5, PCP). Despite these comments, overall, most administrators described PCP engagement as “*a success story*” (OHT25_09, Administrator) and PCPs often agreed that outreach, while ongoing and sometimes challenging, “*has just been really, really positive*” (OHT06_10, PCP).

#### Technology

Information and communication technology were viewed by both PCPs and administrators as either a potential barrier or facilitator of primary care engagement. Most non-PCP participants saw tools such as electronic medical record systems as a facilitator to primary care engagement. As one PFA described, “*You have to have proactive healthcare and enabling with technology… technology is a huge enabler” (OHT 28_4, PFA)*. However, PCPs had two main concerns regarding technology. First, they worried about costs associated with set up and maintenance of a common electronic medical record:

*“…each hospital will come up with their own [EMR solution] and I’d have to tie into three or four apps every day” (OHT27_7, PCP)*

Secondly, PCPs were concerned about data sharing agreements and accountability/responsibility for housing patient health information in a secure manner. This concern was attributed to physicians having been informed that they should not share health information from their health records with other providers except as needed to refer or request care for their patients. Administrators empathized with both concerns and felt that guidance and assistance from the Ministry was needed to address technological integration within the OHT model.

### Theme 3 – ‘We want primary care to be actively involved’: engagement efforts

As the second initiative-level concept, this theme focused on OHT administrators’ efforts to engage primary care. These included methods of outreach and engagement, timing of engagement, and capacity of involvement during the formative phase.

#### PCP outreach and engagement efforts

OHTs often engaged PCP leaders (e.g., executives of primary care health teams) during their formation with the bulk of PCPs following afterwards. These PCP leaders were champions who spearheaded primary care outreach and engagement, seeking buy-in for the approach, which was thought to be beneficial as they “*speak the same language*” (OHT28_4, Administrator) and relate better to the concerns of their peers. Another engagement strategy was to explain how the OHT approach could streamline primary care processes and make PCPs’ roles easier.

*“…my bet is that the OHTs that are able to make it easier for physicians with clients who have complex issues to access both specialist and community services are going to be the ones that the physicians value.” (OHT27_1, Administrator)*

For many administrators, explaining the ‘so what’ proved challenging. To overcome this challenge, they focused on providing novel services such as embedded care coordinators in physician offices and N95 mask fit testing for primary care at the outset of the COVID-19 pandemic. Administrators also emphasized the shared goal of improved patient care, through primary care process efficiencies, which resonated with primary care:

*“This is about patients. And this is only about patients. And so, we just have to keep focused on that.” (OHT15_2, Administrator)*

Outreach methods included developing and circulating written and electronic information materials (e.g., newsletters, surveys), face-to-face visits (e.g., knocking on doors, on-site visits), and hosting meetings (e.g., town halls, symposiums, clinical days). Face-to-face contact at PCP clinics were described as time consuming but successful.

#### Timing of PCP engagement

While OHTs often had a PCP leader early in their formation, most recruited the bulk of their PCPs after the OHT was initiated. There was variability across OHTs as to when primary care should be engaged, whether earlier to assist with the application process or later to assist with clinical decision-making.

*“We didn’t bring them at the steering committee level […] when we were doing the application in the early days, because we didn’t feel that was a good use of their time. We felt that they would feel that they’re adding very little value.” (OHT20_11, Administrator)*

PCPs generally preferred to join earlier as they felt they had a greater voice in the formation process – a desire understood by many administrators and fellow PCP leaders. They explained that they wanted to “*actually be the drivers of change – rather than be mandated change*” (OHT27_10, PCP).

At the same time, some primary care providers were concerned about the (typically uncompensated) demands on their time that early engagement necessitated, as noted below. PFA participants noted the importance of PCPs joining early on as *“primary care is the air traffic controller for anything to do with [patients]” (OHT06_5_PFA)*.

#### Capacity of PCP involvement

PCPs’ roles during the formation of OHTs varied from clinically focused consultants to administrative leaders (e.g., writing the applications). Many OHTs described the value primary care brought to the table and wanted to leverage this value by including PCPs as co-leads or by giving primary care disproportionately large voting voices at the OHT decision-making table.

### Theme 4 – ‘Waiting to hear a little bit more’: primary care concerns about the OHT approach

The last theme describes sectoral-level factors specific to PCPs’ lingering concerns with the OHT model. These included insufficient remuneration, substantial time commitment and inconvenient timing of OHT meetings, adequate representation, maintenance of professional autonomy, and a poor understanding of the impact that the OHT initiative would have on their model of care.

#### Remuneration

Lack of compensation was the most frequently discussed factor seen as impacting primary care engagement across participant types. Physicians described insufficient remuneration as a key factor that impeded engagement.

*“…everyone around the table think[s] physicians are being paid for their time. And that’s without a doubt the biggest barrier that every physician who’s thinking about and/or actively involved with this, worries about and thinks about and gets kind of bitter about to be honest with you…” (OHT06_10, PCP)*

Insufficient remuneration was also discussed by PCPs as a reason for ‘stepping back’ after the OHT applications were submitted. While most administrators understood and sympathized with this concern, some felt that it was unreasonable to be compensating physicians while other contributors, such as PFAs, also remained unpaid.

*“…not only do we need to find a way to compensate family docs and other primary care folks, but we need to figure out a way that is equitable and consistent across OHTs on how we compensate… our client caregivers… it would be unfair.” (OHT16_8, Administrator)*

Administrators and some PCPs reflected on the challenges of equitable compensation for OHT participants given that the Ministry had not yet provided any discretionary funding to the teams.

Of the included cases, the hospital partner of one OHT funded a stipend for primary care involvement in OHT formation activities; however, even with this added support, some PCPs in that OHT felt the compensation was insufficient.

#### Time commitment and timing of meetings

The time commitment and timing of OHT meetings also impacted primary care involvement. PCPs described “*put[ting] in extra hours every night and weekend and abandon[ing] our families and practices*” (OHT07_9, PCP). Scheduling meetings when representatives from all sectors were available was another challenge. PCPs preferred that meetings were scheduled outside of regular business hours, to minimize the impact on their practice; however, administrators and PFAs generally preferred meetings to occur during regular business hours to minimize impact on their personal time. Despite this, administrators understood that the OHT meetings were impeding direct patient care and were therefore contrary to the ethos of the OHT approach:

*“…the whole thing of what we’re trying to do is improve the system for the patient, yet at the same time, this poor physician can’t see his patients because he’s spending so many hours in our meetings.” (OHT24_8, Administrator)*

Indeed, some PCPs described patients threatening to find new care providers due to frequently cancelled appointments or challenges in booking appointments because of conflicting OHT meetings. This challenge was more pronounced for physicians working as solo practitioners and was an often-cited barrier to becoming involved in the OHT during the formational phase.

#### Ensuring representation

PCPs and administrators in all OHTs discussed the importance of balanced sectoral representation as a key factor impacting primary care engagement. Administrators sought to balance the voice of primary care with those of other sectors and patients/caregivers. Conversely, PCPs desired a larger voice to ensure representation from each of the different primary care models of care (e.g., health teams, health organizations, individual practitioners). Due to the number of different primary care models in some OHTs, this balance was hard to achieve.

*“…it became really clear to us that the only way that this is going to succeed is by trying to get a more representative primary care voice to the table to be able to be reflective of the diversity of practice styles and practices in terms of the funding models…” (OHT06_10, PCP)*

A common strategy to address this challenge was to establish primary care groups. These groups varied from informal working groups, physician councils/tables, and primary care networks, to formalized incorporated entities. The creation of groups to unify the voice of primary care was described as: “*…something doctors have never done*” (OHT01_5, PCP) and was considered a value-added outcome of the OHT approach. PCPs felt overwhelmingly positive about the creation of these groups as they were thought to strengthen relationships, trust, and communication between PCPs across models of care. Administrators appreciated these groups as they streamlined engagement with primary care.

#### Professional autonomy

Diminished professional autonomy was a common primary care concern among physicians and nurse practitioners. PCPs were worried about hospital’s delegating tasks, prescribing patient care, and decreasing PCPs’ decision-making authority.

*“The hospital telling you what to do and you’re supposed to do it… I’m feeling uncomfortable with that sort of concept.”* (OHT27_7, PCP)

Administrators did not comment on autonomy.

#### Impact on daily practice

PCPs questioned how OHTs would impact their practice, resulting in a hesitancy to join. They questioned how the OHT approach would impact their funding models (if at all) and their reporting structures (i.e., directly to the Ministry or to the OHT), as an administrator for a Family Health Team explained:

*“You know like I know where I get my funding from, I know what my reporting structure is internally [but] where is [OHT implementation] going to lead to?” (OHT25_5, Administrator)*

The uncertainty around the impact of the OHT kept some primary care physicians on the sidelines as they were “*waiting to hear a little bit about how this impacts them*” (OHT27_3, Administrator). Administrators struggled with this challenge because they felt that they lacked sufficient guidance from the Ministry to adequately address such questions, as explored in Theme 1.

## Discussion

We identified a range of different factors that affected primary care engagement, across initiative-specific, sectoral, and systemic levels during the formation of the OHT integrated care initiative. These factors are related to the limited direction provided by the Ministry, the local context of the OHT (e.g., current state of primary care), and concerns about the OHT efforts by primary care (e.g., renumeration). These factors are interconnected and can either encourage or discourage engagement. There was agreement between PCPs and administrators on many of these factors; however, some key differences were identified.

Some participants in our study valued PCP participation in the formative stages of the OHTs because they felt that changes to care pathways were likely to start with primary care. While OHT leadership included PCPs who shaped engagement efforts and strategic priorities, broad scale PCP recruitment generally occurred after OHT formation. The literature supports early engagement of key stakeholders as those directly affected by a change should be involved in the change process to improve acceptability and, ultimately, the success of the change [25,26]. Some administrators in our study demonstrated the value they placed in primary care engagement through early primary care involvement in system co-design and by encouraging PCPs to lead or co-lead the OHT. Shared leadership has been identified as a core enabling factor for integrated healthcare according to two recent reviews [26,27]. It will be of interest to follow the OHTs over time to observe how PCP involvement will impact the success of the OHT.

In contrast to administrators, physician leaders seemed more wary of the OHT approach. Many agreed that an integrated approach to care would benefit patients and could break down silos and improve communications across sectors. Such efficiencies were valued by PCPs, and are generally thought to improve engagement and the success of a new initiative [25]. However, PCPs described concerns around remuneration and professional autonomy, which they felt were at odds with the current OHT approach. Participants in our study suggested that all OHT participants, including PCPs, should be compensated for their time due to the shared understanding of the time commitment needed to participate. Administrators struggled with how to adequately compensate PCPs in an equitable way with other OHT partners (e.g., PFAs) and generally seemed unaware of PCPs’ concerns around professional autonomy. Adequate compensation has been highlighted in the implementation literature generally, as well as specifically to integrated care, as key to success [26,27,28]. Although the Ministry announced funding for approved teams, at the time of this study, during the formational stage, no funding was provided. Further, our participants explained that compensation should be the same across the OHTs to avoid providing (dis)incentives for PCPs to join one OHT over another. Clear delineation of responsibilities and accountabilities have also been recommended, for example, through formal accountability agreements [26,27], which could alleviate PCPs’ concerns around diminished professional autonomy. In contrast, there was agreement among PCPs and administrators about the importance of addressing local context, such as cross-sectoral relationships and experiences with previous integrated care initiatives, when engaging with PCPs in OHT formation. Without considering these factors, the success of the OHT approach could be threatened.

Our findings highlight the need for broad primary care involvement (beyond physician leaders) and engagement efforts made by OHTs. Engagement efforts should account for different levels and inter-relations of factors. In the case of this study, these emerged at health system, integrated care initiative, and sectoral levels. For example, the Ministry’s decision to encourage grassroots strategies and solutions (at the health system-level) challenged OHTs’ ability to articulate the relative advantages [25] for primary care involvement in OHTs during their outreach and engagement efforts (integrated initiative-level), which in turn increased PCP concerns around impact on their practice (sectoral-level). The factors in each of these three levels affect primary care engagement either by improving involvement and buy-in or by decreasing engagement, leading to frustration, disenfranchisement, and ultimately, disengagement. While frameworks exist for enabling integrated care [27,29] that include primary care concepts [30], to the best of our knowledge, and after consulting an information specialist, they do not specifically address primary care engagement. Our findings (e.g., the interrelated levels of factors) could therefore inform future research aimed at developing primary care engagement frameworks and guide future implementation research on factors that may influence integrated care.

### Strengths, Limitations and Future Research

We sought a diversity of perspectives by purposefully including OHTs with variation in leadership and geography. However, we had limited specialist physician, nurse practitioner, or other front-line provider participants from community-based organizations due to the structure of current OHT models themselves. Future studies should include these perspectives which contribute substantially to implementation of community-based integrated care initiatives. Our research provides a snapshot of factors affecting primary care engagement during the early formation of OHTs and future research should seek to understand if and how these factors change over time as OHTs mature. Further, perspectives from PCPs who choose not to participate in an OHT should be explored.

## Conclusion

A multitude of system-level, initiative-level, and sectoral-specific factors affected primary care engagement during the formation of OHTs. These factors are interconnected across levels and should be considered when engaging with PCPs within an integrated care approach. Future research may want to focus on integrated care delivery initiatives as they mature, in order to address historical gaps in the involvement of primary care physicians.

## Additional File

The additional file for this article can be found as follows:

10.5334/ijic.5704.s1 Appendix A. Interview Guide.
