# Supplementary material for: Factors Impacting Primary Care Engagement in a New Approach to Integrating Care in Ontario, Canada
Source: Int J Integr Care. 2022 Mar 4;22(1):20. doi: 10.5334/ijic.5704 (PMC8896242; doi:10.5334/ijic.5704)
Supplement: Appendix A. — Interview Guide. [file ijic-22-1-5704-s1.pdf]

Appendix A: Interview Guide

Role

1. Please describe your position and role a) within your organization b) within the OHT.

Preparation & Objectives

2. What is your vision for your OHT?
3. In your opinion, why did [organization] want to become part of an OHT?
    a. Does [organization] have previous experience with integrated care initiatives?
4. Can you describe what [organization] has done in preparation for OHT formation?

Partnership & Leadership

5. How did partner organizations come together?
6. We're interested in understanding how partners across organizations work together.
    a. How would you describe the level of collaboration across organizations?
    b. How have decisions been made so far?
    c. What are some of the key differences between partner organizations? Do these differences challenge your ability to work with each other? How are they negotiated?
    d. Is there a story or anecdote that comes to mind that captures what working together has been like for you?
7. In your opinion, who have been the most important people driving forward the OHT model?
    a. Have they been effective leaders? What has made them effective?
    b. Is there a lead organization? What is its role?
    c. Has the OHT faced any leadership-related challenges?

Model & Pathway

8. [This OHT] chose to initially focus on [population/s]. How was this patient population identified?
    a. What are some of the key considerations informing pathway design?
9. Do you think the OHT will make a difference to the organization and delivery of care?
    a. What differences will a) clinicians and b) patients experience?
10. How is care coordinated?
    a. Is care coordination done differently for the OHT, compared to how it was before?
    b. Could you tell me what care coordination would look like to you in an ideal world? How do your plans for care coordination compare?

Patient Engagement

For non-Patient Family Advisors (PFAs):

11. Are you satisfied with the extent to which patients/ caregivers have been involved in your OHT?
    a. What was done well? What challenges remain?

For PFAs:

12. As a patient/caregiver partner, have you felt able to contribute to the OHT? How have you done so?
13. Do you feel that the OHT adequately accounts for PFA perspectives?

Clinician Engagement

14. Were clinicians included during OHT development? What did engaging them involve?
    a. How receptive have clinicians/ physicians been to the OHT initiative?
    b. Are you satisfied with the level of engagement of primary care? What remains to be done?

Communication/ Clinical Information Sharing

15. How do clinicians typically share information about patients? Are there plans to change processes/ systems?
    a. What i) are your key challenges, ii) is going well, as it relates to data-sharing?

Financial Matters

16. Will there be any changes in funding/ accountability structures under the OHT model?
    a. Will the OHT initiative require any additional resources (e.g., for new roles, systems)?

Evaluation

17. What do you think are the most important things to evaluate in relation to the OHT model?

Reflection

18. What have been your greatest challenges/ main sources of resistance? How has the team negotiated this?
19. Is there anything specific to your geography, patient population, etc. that requires you to implement the OHT differently from what is envisaged by the Ministry?
20. Is the OHT approach a promising model? A sustainable one?
21. Is there anything you would like to touch on that we have not spoken about?
